# A Bayesian approach to investigate life course hypotheses involving continuous exposures

**DOI:** 10.1093/ije/dyy107

**Published:** 2018-06-14

**Authors:** Sreenath Madathil, Lawrence Joseph, Rebecca Hardy, Marie-Claude Rousseau, Belinda Nicolau

**Affiliations:** 1 Faculty of Dentistry, McGill University, Montreal, QC, Canada; 2 Epidemiology and Biostatistics Unit, Institut Armand-Frappier, INRS, Laval, QC, Canada; 3 Department of Epidemiology, Biostatistics and Occupational Health, McGill University, Montreal, QC, Canada; 4 MRC Unit for Lifelong Health and Ageing at UCL, University College London, London, UK

**Keywords:** Bayes theorem, epidemiological method, life course

## Abstract

**Background:**

Different hypotheses have been proposed in life course epidemiology on how a time-varying exposure can affect health or disease later in life. Researchers are often interested in investigating the probability of these hypotheses based on observed life course data. However, current techniques based on model/variable selection do not provide a direct estimate of this probability. We propose an alternative technique for a continuous exposure, using a Bayesian approach that has specific advantages, to investigate which life course hypotheses are supported by the observed data.

**Methods:**

We demonstrate the technique, the relevant life course exposure model (RLM), using simulations. We also analyse data from a case-control study on risk factors of oral cancer, with repeated measurements of betel quid chewing across life. We investigate the relative importance of chewing one quid of betel per day, at three life periods: ≤20 years, 21–40 years and above 40 years of age, on the risk of developing oral cancer.

**Results:**

RLM was able to correctly identify the life course hypothesis under which the data were simulated. Results from the case-control study showed that there was 74.3% probability that betel quid exposure earlier in life, compared with later, results in higher odds of developing oral cancer later in life.

**Conclusions:**

RLM is a useful option to identify the life course hypothesis supported by the observed data prior to the estimation of a causal effect.

**Key Messages**

- Current strategies to investigate life course hypotheses have shortcomings, and analytical techniques to accommodate the hierarchical nature of life course hypotheses are warranted.
- We demonstrate the strength of Bayesian inference to directly estimate the posterior probabilities of different life course hypotheses.
- We propose a novel model to analyse life course hypotheses, which circumvents several shortcomings of previous approaches.

## Introduction

Life course epidemiology aims to understand the long-term effects of exposures that occurred during different life periods, particularly their effects on adult health and disease.^1^^,^^2^ To be able to test these effects, Ben-Shlomo and Kuh proposed a typology of life course models that are broadly classified into critical/sensitive period and accumulation/chains of risk.^1^ More recently, the hierarchy among life course hypotheses and need for statistical techniques that account for this hierarchy have been recognized.^3^^,^^4^

The development of analytical techniques to identify the life course hypothesis that best describes the exposure-outcome relationship is a constantly evolving field. The structured approach for a binary exposure, proposed by Mishra *et al*., has attracted considerable interest.^5–8^ The method compares a set of nested models, each capturing one life course hypothesis, with a saturated model. The goodness-of-fit statistic is used to select the nested model that shows statistically non-separable model fit compared with the saturated model.^9^

Although this approach is useful, there are conceptual and pragmatic concerns. First, it assumes that there is one true model among the tested models, whereas in reality the data may result from a mixture of models. In addition, this structured approach is limited by the influence of sample size on *P*-values. For example, situations may exist in which all life course hypotheses have *P*-values greater than a threshold value, due to small sample size. Conversely, in studies with large sample sizes, all nested models might have *P*-values lower than the threshold. Moreover, when more than one nested model fit the data equally, the findings from these models may contradict each other. Also, model selection based on two different criteria (F-test, Akaike Information Criterion) may result in the identification of different models. This uncertainty in model selection is not reflected in the estimates of the selected model. Furthermore, the number of nested models increases as the number of life periods to test increases. This results in multiple testing and requires strategies to control type 2 error inflation.

Extending Mishra *et al*.’s model to continuous exposure measurement is challenging, as the number of parameters in the saturated model may be close to the sample size. Recently, Smith *et al*. suggested a modification to Mishra *et al*.’s structured approach, using least angle regression (LARS) for both continuous and binary exposure measures under a linear regression model.^10^^,^^11^ In their simulation studies for binary exposures, the LARS method outperformed both the F-test and AIC-based structured approach in most of the scenarios tested, except for highly correlated exposure measures and the sensitive period model. However, the performance of the LARS method for the structured approach has not been tested for binary outcome measures, a common scenario in life course epidemiology of chronic diseases. In addition, this method does not consider the hierarchical nature of life course hypotheses and includes model selection. Hence, there is a need to develop a strategy that can accommodate a wide variety of exposure and outcome variables and considers the hierarchy among life course hypotheses.

**Figure 1.**
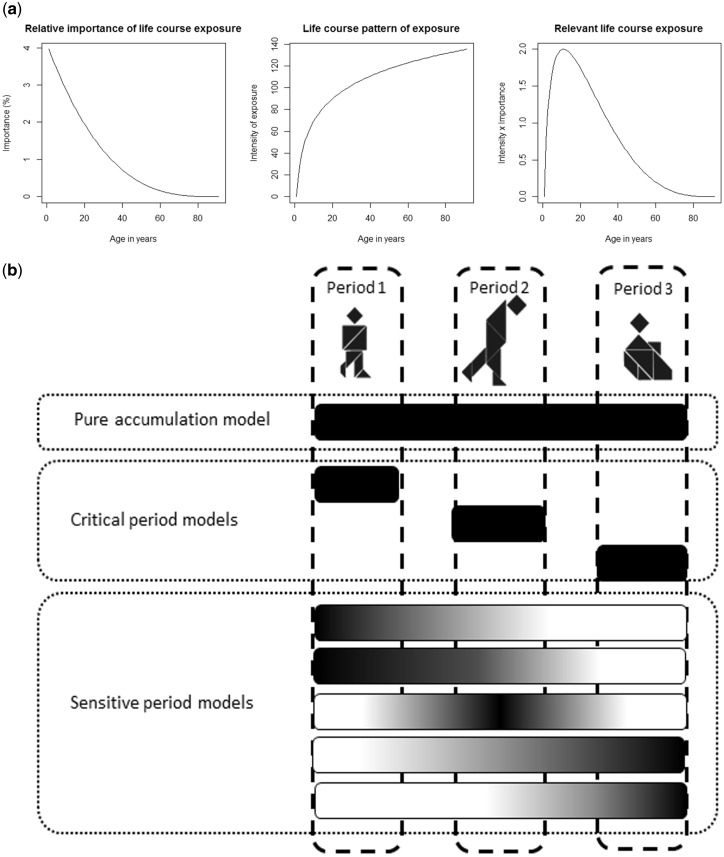
Life course conceptual models of the relative importance of exposures at different periods in relation to adult health or disease. 1(a). Illustrated example for protracted exposures. 1(b). Example with three life periods (childhood, early adulthood, late adulthood). Gradients represent examples of the relative importance of life course exposure, white indicating lowest importance and black indicating highest importance.

In the case of a single exposure measure, testing life course models is an investigation of the relative importance of the exposure in different periods of life in relation to an outcome (Figure 1a). In this context, the accumulation hypothesis (periods have the same importance) and critical period hypothesis (only one period is important) could be seen as special cases of a more general sensitive period hypothesis (different periods have different importance).

We propose a modelling strategy to test life course hypotheses with continuous exposures: the relevant life course exposure model (RLM). This technique: (i) does not require model/variable selection; (ii) incorporates the hierarchical nature of life course hypotheses; (iii) can be used for both continuous or categorical outcome variables; (iv) can accommodate missing values and measurement errors.

## Relevant life course exposure model

The RLM assumes a weight for the exposure experienced during each life period. The weight relates to the relevance of exposure during that period to the development of the outcome of interest later in life. Then, the relevant life course exposure is conceptualized as the product of the exposure metric and its corresponding weight over each life period, summed over all life periods.

The relevant life course exposure varies according to the different life course hypotheses. For example, under a critical period hypothesis, the relevant life course exposure is entirely constituted of exposure in one period that is considered critical for the later life outcome. By contrast, under a sensitive period hypothesis, the exposure during periods that are sensitive will be allocated greater weights compared with other periods. Finally, in the pure accumulation hypothesis, exposure in every period has equal weight. Figure 1b illustrates these concepts using three life periods.

Because in RLM the values of the weights inform the life course hypothesis, we estimate them from the data. In addition, we also estimate the lifetime effect of the exposure, that is the overall effect of relevant exposures accumulated over a person’s lifetime. Similar weighting systems have been proposed in other contexts.^12–14^ In this article, we describe our adaptation and expansion of this method to life course epidemiology using a Bayesian approach. We show its applicability to epidemiological research employing simulations and an analysis of real-life data. We also compare our method with the structured approach using simulated data. Finally, we discuss how to include prior knowledge into the analysis and make inferences on the life course hypotheses given the data.

## Methods

Let t=1, 2, 3,…,T denote the temporal ordering of the repeated measurements of the exposure, $\;x_{ti}$, be the exposure measurement at time t, and $y_{i}$ be the outcome of interest measured at the last time point, for the $i^{th}$(i=1, 2,…, N) participant. The time scale (t) could be age at exposure or denote particular life periods (e.g. childhood, adolescence and adulthood). The weight for exposure at time t is modelled as an arbitrary function$\;(w_{t}=f\left(t\right))$; it is assumed that each weight takes a value between 0 and 1 and that the sum of all weights is equal to 1. The relevant life course exposure is then defined as the weighted sum of exposures in all time periods (Equation 1):

(1) $$lx_{i}=\sum_{t=1}^{T}w_{t}\;*\;x_{ti}\;$$

Subsequently, the association between the relevant life exposure variable and the outcome $y_{i}$ could be modelled in a generalized linear model framework, $g\left\{E\left(y_{i}\right)\right\}=\mu_{i};\;where\;g\left\{.\right\}$ is a link function, as below:

(2) $$\;\mu_{i}=\beta_{0}+\delta \;*\;lx_{i}+\lambda \;*\;C_{i}$$

where, δ is the lifetime effect for the exposure. *Lambda*(λ) is the column vector of coefficients for the covariates $C_{i}=(c_{1i},\;c_{2i},\;\ldots ,\;c_{pi})$.

To fit the RLM, we use a Bayesian approach that requires the specification of prior probability distributions (often referred to as ‘priors’) on the weights, the lifetime effect of the exposure and other unknown parameters in the model.

## Stating priors for life course hypotheses

**Figure 2.**
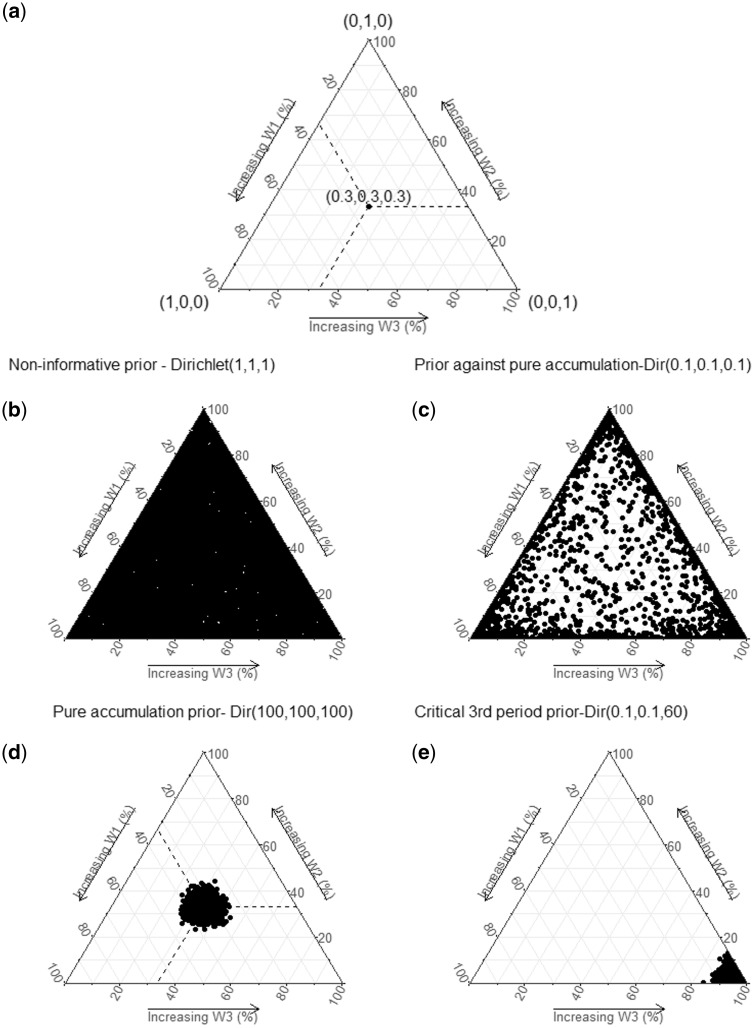
Examples of multivariate priors for weights in Bayesian relevant life course exposure models. 2(a). The three sides of the triangle plots represent three weights in percentage, one for each life period considered. The vertices represent corresponding extreme weights [(1, 0, 0), (0, 1, 0), (0, 0, 1)] and the midpoint of the plot represents equal weights (0.33, 0.33, 0.33). The concentration of dots represents the density of the corresponding Dirichlet distribution at that point. 2(b) illustrates equal distribution of density (equal probability for any combination of weights). 2(c) illustrates higher probabilities at the vertices compared with midpoint (favours critical period compared with accumulation hypothesis). 2(d) illustrates higher probabilities at the midpoint, favouring a pure accumulation hypothesis (w1 = w2 = w3). 2(e) illustrates higher probability at one vertex (favouring critical period at one vertex; w1 = 0, w2 = 0, w3 = 1).

In Bayesian RLM, priors on the weights can be used to express the level of uncertainty about the life course hypothesis behind the data generating process. Because the weights are modelled as a unit-simplex, a Dirichlet distribution (Dir) is the natural choice for a conjugate prior. Alternatively, priors can be placed on marginal distributions of weights (e.g. Beta distributions). Figure 2 shows examples of Dirichlet prior distributions for the weights using three life periods. For instance, a non-informative prior on weights for the three life periods can be specified jointly as Dir(1, 1, 1) (Figure 2b) or Beta(1, 1) for each weight separately. Both of these distributions specify equal densities over supported values. Such non-informative priors are particularly useful if the goal is to identify the life course hypothesis supported by the data alone (see below). As is generally the case with Bayesian analysis, if the data have sufficient information to update the parameters, the choice of distribution for non-informative priors will only have a trivial effect on the results.

An informative prior favouring the critical period over the accumulation hypothesis could be stated as a Dirichlet distribution with higher densities along the vertices (Figure 2c), whereas a prior favouring the accumulation hypothesis could be stated as a Dirichlet distribution with a higher density at the centre (Figure 2d). A Dirichlet prior with a relatively higher density near one vertex could be used to state prior evidence of a critical period hypothesis (Figure 2e).

## Identifying the life course hypothesis supported by the data using Bayesian RLM

In Bayesian RLM, the posterior distribution of weights conditioned on a non-informative prior can be used to identify the life course hypothesis supported by the data. A measure of the difference between the estimated and expected weight vectors (e.g. Euclidean distance) can be used for this purpose (Table 1).^15^ The shortest Euclidean distance identifies the life course hypothesis most supported by the data. However, the true situation may result from a combination of different life course hypotheses and this method can identify the correct ‘mixture’ of them. In the next section, we demonstrate the Bayesian RLM using simulated and real-life data examples.

**Table 1.** Three main life course models and corresponding weight functions

Life course model	Weight function
Pure accumulation model	$w_{1}=w_{2}=w_{3}=\frac{1}{3}$
Critical period models	$w_{1}=1\;and\;w_{2}=w_{3}=0$
	$w_{2}=1\;and\;w_{1}=w_{3}=0$
	$w_{3}=1\;and\;w_{1}=w_{2}=0$
Sensitive period models	$0<w_{t}<1,\;for\;t=1,\;2,\;3\;and\;$$w_{1}>w_{2}\;and\;w_{3}$
	$0<w_{t}<1,\;for\;t=1,\;2,\;3\;and\;$$w_{2}>w_{1}\;and\;w_{3}$
	$0<w_{t}<1,\;for\;t=1,\;2,\;3\;and\;$$w_{3}>w_{1}\;and\;w_{2}$

t denotes the time points of measurements.

### Simulation study

The objectives of the simulation study were: (i) to assess whether the RLM estimates the ‘true’ values for the parameters (the weights $\left[w_{t}\right]$ and lifetime effect [δ]), and hence, identifies the life course hypothesis correctly; (ii) to assess the performance of the model under different sample sizes using absolute bias; (iii) to compare the method with Mishra *et al.*’s structured approach.

For simplicity, we simulated a three-period life course study assuming no measurement error in the variables. We simulated three Gaussian exposure variables with a correlation of 0.7 and 0.49 between adjacent and non-adjacent measures, respectively. A binary dependent variable was simulated using a logistic likelihood function. Datasets were simulated for all combinations of the three life course hypotheses and three sample sizes (*n* = 700, 1500, 3000). The hypotheses and corresponding weight values were: (i) third life period as a critical period [w1 = w2 = 0 and w3 = 1]; (ii) pure accumulation hypothesis [w1 = w2 = w3 = 0.333]; and (iii) first life period as a sensitive period [w1 = 0.75, w2 = 0.20, w3 = 0.05].

Bayesian RLM using a logistic regression were fitted applying a non-informative Dirichlet prior for weights [W∼Dirichlet(1, 1, 1)] and a weakly informative Cauchy prior on the lifetime effect$\;[\;\delta ∼\;Cauchy\left(0,\;2.5\right)]$. The Cauchy distribution was chosen because its fatter tails allow more support for extreme values compared with a normal distribution. For each dataset, we ran four parallel Hamiltonian Monte Carlo chains and considered the first 25 000 iterations for burn-in and subsequent 25 000 iterations for inference. Convergence was assessed using trace plots and Rhat values.^16^^,^^17^ We plotted the posterior distributions of weights and the lifetime effect of the exposure along with their 95% credible intervals (95% CrI). We also computed Euclidean distances from five reference weight vectors (one accumulation, one sensitive period and three critical period hypotheses) to the estimated values of weights.

We considered that the Bayesian RLM correctly identified the life course hypothesis if the shortest Euclidean distance corresponded to the ‘true’ life course scenario and the 95% CrI for estimated weights included the true parameter value.

### Comparing Bayesian RLM with the structured approach

We compared Bayesian RLM with the structured approach suggested by Mishra *et al.*,^9^ using the simulated dataset described above. As previously indicated, the saturated model used in the structured approach cannot be estimated for continuous exposures, and therefore we cannot use the likelihood ratio test to select the best model. Hence, we compared these approaches from a Bayesian perspective using the Watanabe-Akaike information criterion (WAIC) where the model with the lowest WAIC was selected in the structured approach.^18–20^ We then compared the WAIC for the selected model from the structured approach with the WAIC for the Bayesian RLM model.

### Real-life data example: life course betel quid chewing and risk of oral cancer—HeNCe Life Study—India

We used data from the Indian arm of the HeNCe Life study, an international hospital-based case-control study investigating life course risk factors of head and neck cancers (HNC) in three countries (India, Canada and Brazil), as previously described.^21–23^ Briefly, during the period 2008 to 2012, 350 participants with histologically confirmed, primary squamous cell carcinoma of the oral cavity were recruited from two tertiary care centres in Kozhikode, South India. Non-cancer controls (*n* = 371) were recruited from the same hospital as cases and frequency-matched by age (5-year groups) and sex to cases using incidence density sampling. An array of exposures throughout participants’ lives were measured with the help of life grid-based structured interviews, which have good reliability for retrospective life course data collection.^24^^,^^25^

Betel quid chewing, a popular smokeless tobacco habit in South-East Asia, is a known risk factor for oral cancers.^26^^,^^27^ In the current analysis, we investigate the relative impact of this habit during three life periods (0–20, 21–40 and above 40 years of age) on the risk of developing oral cancer later in life. Chew-years, the unit of exposure in each period, corresponds to chewing one betel quid per day for 1 year.^21^

We modelled the weights and lifetime effect hierarchically, allowing these parameters to vary by age group at interview (below 40, 41–50, 51–60, 61–70, above 70 years of age) centred around an average value for the population (details in Supplementary data are available at *IJE* online). This stems from the hypothesis that participants from different birth cohorts may have lived diverse experiences. For participants below 40 years of age, the weight for the last period (>40 years) was constrained to be zero, and the first two period weights constrained to sum to one.

Age at interview, sex, pack-years of tobacco smoking, education, lifetime consumption of alcohol (litres of ethanol) and a material deprivation index were considered as potential confounders. Continuous variables among potential confounders were rescaled to Z-scores to improve mixing of Markov Chain Monte Carlo (MCMC) chains. To account for the qualitative difference between an ever user of betel quid (at least 1 year of use before interview) and a never user, a binary indicator variable was included in the model. With this strategy, the lifetime effect [δ] can be interpreted as the effect of the relevant life course exposure of betel quid chewing among ever users only. There were 37 (5.1%) participants with missing values in the material deprivation variable. To account for the uncertainty in the missing information, we performed a full Bayesian imputation of missing values. Because this variable represents the number of items possessed out of 34 indicators of deprivation,^28^ a binomial regression model was used for imputation. We used non-informative priors for the hyper parameters and a weakly informative prior on all other parameters (details in Supplementary data are available at *IJE* online).

## Results

### Simulation

**Figure 3.**
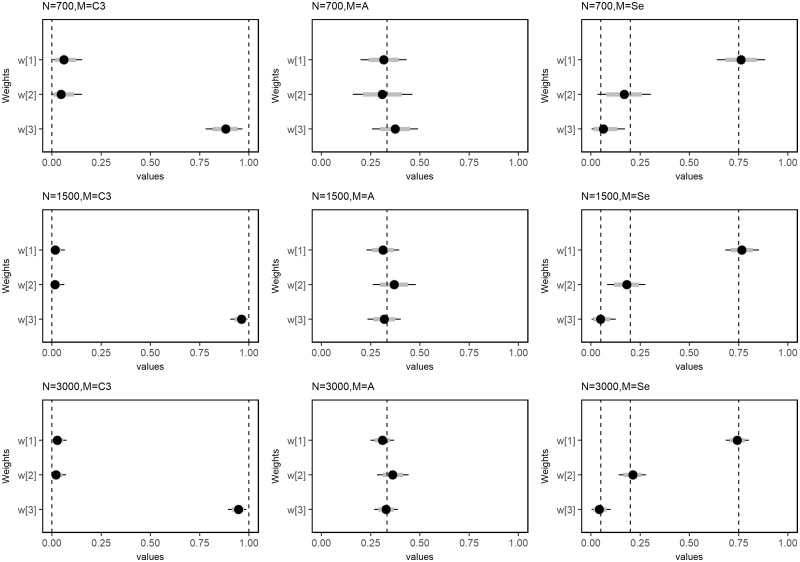
Mean and 95% credible intervals of posterior distributions of weights under three life course scenarios and sample sizes. The dashed vertical lines denote the true parameter values: first column—critical period scenario (w1 = 0, w2 = 0, w3 = 1); second column—accumulation scenario (w1 = w2 = w3 = 0.33333); third column—sensitive period scenario (w1 = 0.75, w2 = 0.20, w3 = 0.05). The grey horizontal bars represent 80% posterior credible intervals and horizontal solid line represents the 95% credible intervals. N—sample size, Model—life course scenario.

**Figure 4.**
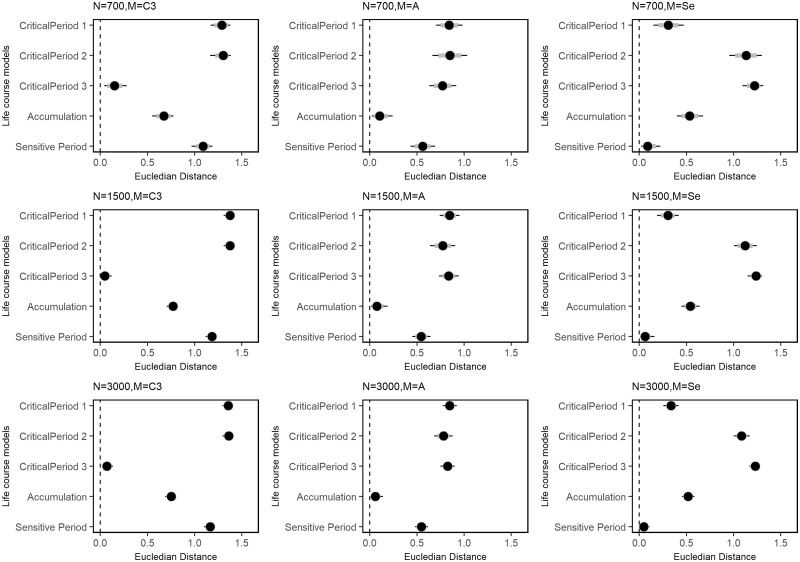
Means and 95% credible intervals of posterior distributions of Euclidean distances under three life course scenarios and sample sizes. Columns represent the life course scenario under which data were simulated and rows represent different sample sizes. The Y-axis of each plot shows the following reference vectors to estimated weights: CP-1 (w1 = 1, w2 = 0, w3 = 0); CP-2 (w1 = 0, w2 = 1, w3 = 0); CP-3 (w1 = 0, w2 = 0, w3 = 1); A (w1 = w2 = w3 = 0.3333); SP (w1 = 0.75, w2 = 2.0, w3 = 0.05).

**Figure 5.**
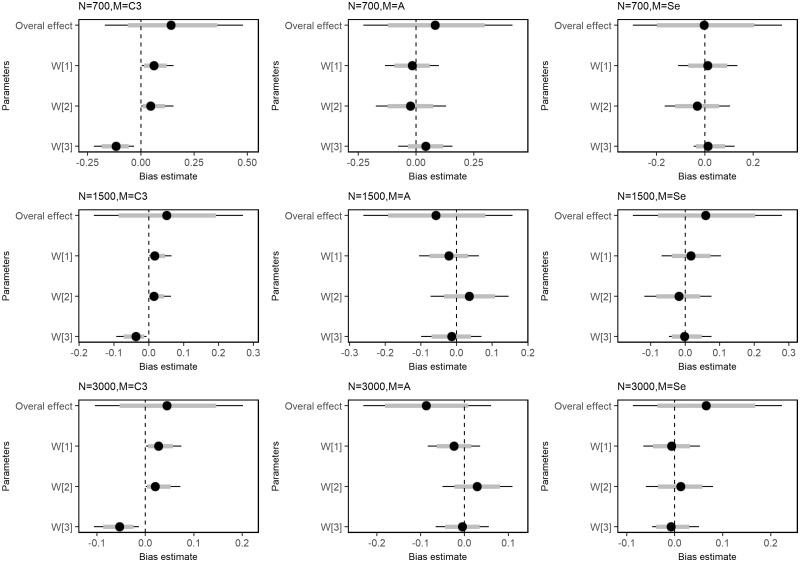
Means and 95% credible intervals of posterior distributions of bias from true parameter values under three life course scenarios and sample sizes. Columns represent the life course scenario under which data were simulated and rows represent different sample sizes. The Y-axis of each plot shows the following reference vectors to estimated weights: CP-1 (w1 = 1, w2 = 0, w3 = 0); CP-2 (w1 = 0, w2 = 1, w3 = 0); CP-3 (w1 = 0, w2 = 0, w3 = 1); A (w1 = w2 = w3 = 0.3333); SP (w1 = 0.75, w2 = 2.0, w3 = 0.05).

The Bayesian RLM correctly identified the life course hypothesis under which the data were simulated for all sample sizes studied. Figures 3 and 4 display the posterior mean and 95%CrI of estimated weights and the Euclidean distances. Estimates from the smallest dataset (*n* = 700) had higher absolute bias compared with others (Figure 5 and Supplementary Table 2, available as Supplementary data at *IJE* online). Although Bayesian RLM provided biased estimates of weights under a critical period scenario, the identification of the correct hypothesis was not affected by this bias (Supplementary Tables 1 and 2, available as Supplementary data at *IJE* online). The 95% CrI for the lifetime effect [δ] included the true parameter of the lifetime effect, with which data were simulated, for all combinations of sample sizes and scenarios tested.

The structured approach to model selection provided the same inference as Bayesian RLM. In the structured approach, the model corresponding to the true hypothesis had the lowest WAIC value for each life course hypothesis. The WAIC of the Bayesian RLM was close to the lowest WAIC model from the structured approach (Supplementary Table 3, available as Supplementary data at *IJE* online).

### Real-life data example: HeNCe Life study

**Figure 6.**
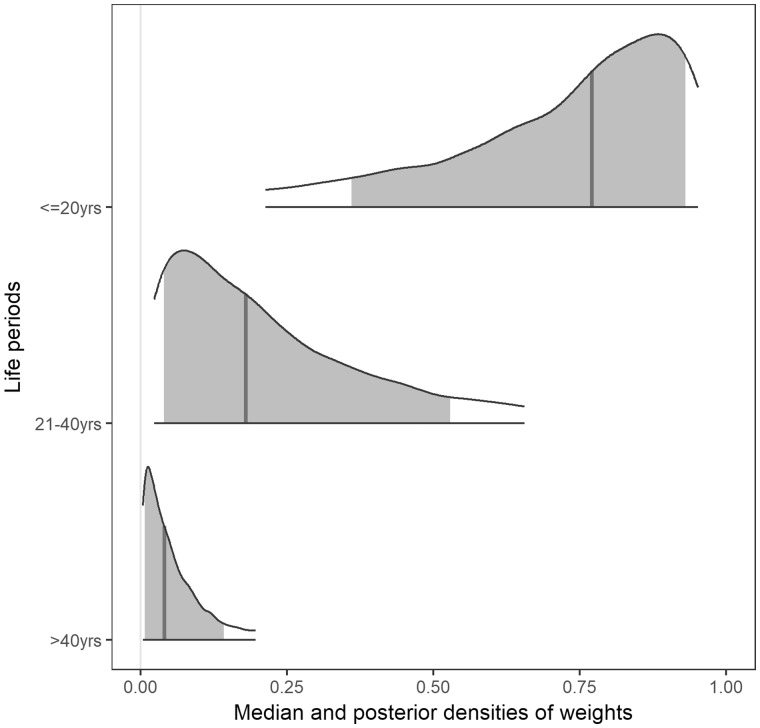
Marginal posterior densities of weights (average weights across age groups), estimated for exposure to betel quid chewing during three life periods for the risk of developing oral cancer. The vertical solid line represents the median, and the shaded area represents the 80% credible interval.

**Figure 7.**
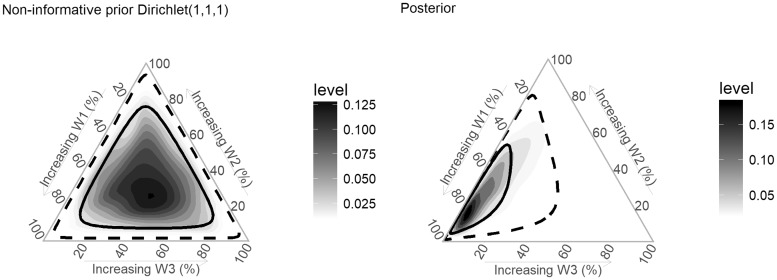
Densities and credible limits of prior and posterior joint distributions of weights for exposure to betel quid chewing during three life periods with regard to risk of developing oral cancer. Darker areas represent higher densities. The solid line represents the 50% credible limit and the dashed line represents the 90% credible limit.

Life course exposure to betel quid chewing across age groups showed variation; older age groups had relatively higher average amounts of chew-years at each life period (Supplementary Table 4, available as Supplementary data at *IJE* online). The Bayesian RLM results showed evidence for a sensitive period with exposures earlier in life having greater relevance in all cohorts (Figures 6 and 7 and Supplementary Figure 1 and Table 5, available as Supplementary data at *IJE* online). On average, the amount of betel quid chewed in the age periods ≤20, 21–40 and >40 years contributed 70.2%, 23.6% and 6.1%, respectively, to the relevant life course exposure (Table 2). Among betel quid users, there was 85.3% posterior probability for the hypothesis that 20 years and younger is a sensitive period for betel quid exposure compared with later life periods (w1 > w2 and w3) for the risk of oral cancer. In addition, there was 74.3% posterior probability that betel quid exposure earlier compared with later in life (w1 > w2 > w3) results in higher odds of developing oral cancer.

**Table 2.** The relative importance of betel quid chewing exposure at different life periods for the risk of developing oral cancer, HeNCe Life study, India

Life periods (age, in years)	Mean (%)	Median (%)	95% credible interval (%)
≤20	70.2	77.1	10.3–96.4
21–40	23.6	17.9	1.4–75.8
>40	6.1	4.0	0.2–24.7

Among ever users of betel quid, one chew-year increase in life course cumulative exposure to betel quid increased the odds of developing oral cancer by a factor of 1.04 [odds ratio (OR) = 1.04, 95% CrI = 1.00–1.15] (Supplementary Table 7, available as Supplementary data at *IJE* online).

## Discussion

The pioneers of life course epidemiology, Kuh and Ben-Shlomo, have recently identified the need to investigate life course hypotheses by considering them as special cases of an all-inclusive model rather than as separate hypotheses.^3^^,^^4^^,^^29^ Current life course analytical strategies do not take into account this hierarchy and rely on *P*-values. To address this shortcoming, we propose the Bayesian relevant life course exposure model which identified the correct life course hypotheses in our simulation studies. We further demonstrated the method using a real-life data example from oral cancer research. Betel quid chewing earlier in life, compared with later, resulted in a higher risk of developing oral cancer.

### Latency and life course effects

As cancers of other anatomical sites, oral cancers have a latency period. The life course effect is influenced by the latency of disease, age-dependent vulnerability and social factors that determine the life course trajectory of exposures. For example, exposures occurring during life periods closer to the average age of cancer incidence in a population may not be important for the disease risk due to the latency of cancer. This might result in evidence for a sensitive period earlier in life, even without any ‘true’ age-dependent effect. Although disentangling the latency effect from the ‘true’ life course effect may be challenging, a step toward this goal is to test the life course models in different age groups. For instance, in our real-life data example we performed a hierarchical Bayesian RLM in which different age groups were allowed to follow different life course hypotheses. However, all age groups showed a sensitive period during the first 20 years of life.

### Bayesian RLM for protracted exposures with closely spaced measurements

In life course studies with an exposure measured at multiple closely spaced time points, estimating weights for each period separately may require sample sizes that are not achievable. One solution is to assume a parametric shape, as in latency models, to arbitrarily weigh the function in Equation 1.^30^ However, this method is not suitable in the life course setting where the shape of the weight function is of particular interest. Although not specific to life course research, a similar weighting of exposure approach has previously been used assuming a B-Spline with prior specification of the number of interior knots and degree of spline.^12^ Sylvestre *et al.*^14^ further extended this idea to proportional hazard models, and used a model selection procedure based on the BIC to choose the number and position of the knots for the B-Spline. The overall effect and the weight function were combined into a single parameter to increase identifiability and ease of fitting the model in standard statistical software. An extension of this method has recently been developed, in which the nonlinear effect of exposure at each time point may also be estimated.^31^^,^^32^ Alternatively, a time window-specific analysis has been proposed using a Bayesian hierarchical approach to allow deviations from a parametric latency function.^33^ Our approach differs from these methods by directly estimating the weight function and subsequent probability of life course hypotheses, which is of interest in life course epidemiology.

Techniques that aim to extract parameters, which describe individual exposure trajectories, and then relate these to outcomes have been proposed in life course epidemiology. Super imposition by translation and rotation (SITAR) models^34^ and random effect models using splines^35^ are examples of such models, particularly used in studies investigating how childhood growth is associated with adult health. An extension of the SITAR model to multivariate surfaces (involving trajectories of more than one life course variable) has also been proposed.^36^ All of these techniques provide effect measures for deviations from the average trajectory of exposure. For example, McCarthy *et al*. estimated that the velocity of growth from 1 year and 9 months to 5 years was the strongest predictor of adult BMI.^37^ Compared with these approaches, Bayesian RLM facilitates direct inference on life course hypotheses of critical, sensitive periods and accumulation.

RLM can be extended to exposure measurements made in closely spaced windows by using a summary of exposures (e.g. averages, cumulative sum) in wider age strata. However, the choice of cut-off points to define the age strata may affect the results. For example, if the true critical or sensitive period is divided between two strata, the corresponding weights will be distributed across these strata. Stone *et al*. have investigated how to best define age strata in life course studies, and found that theory-driven categories have superior properties compared with data-driven approaches.^38^

Alternatively, RLM could be extended to estimate the shape of the weight function using Bayesian adaptive priors for the splines,^39^^,^^40^ with the advantage of not having to perform model selection.

### Advantages and limitations of Bayesian RLM

Bayesian RLM can directly estimate the probabilities that different life course hypotheses are true, conditioned on the observed data and prior beliefs. In the example above, we estimated the posterior probability of the hypothesis that people chewing betel quid earlier in life have a higher risk of developing oral cancer.

The Bayesian approach in RLM also provides the flexibility of simultaneously estimating the weights and the lifetime effect, without the need for specialized estimators. For example, it is relatively easy to impose inequality constraints on parameters (e.g. weights add up to 1) in a Bayesian approach compared with the classical statistical approach.^41^^,^^42^

The Bayesian approach offers additional advantages such as the ability to formally include prior knowledge, easily accommodate missing values, and incorporate the hierarchical nature of data and parameters.^43–48^ Both the structured approach^9^ and the LARS approach^10^^,^^11^ require additional steps to account for uncertainty associated with missing information on covariates. As demonstrated in our example, in Bayesian RLM the uncertainty in missing information can be propagated to the inference on life course hypotheses by including an imputation model.

In the Bayesian approach, prior distributions can be used to transparently incorporate theory into the analyses. As demonstrated in our example, the incorporation of theory is not limited to prior distributions. For example, there could be a cohort effect for the relative importance of an exposure at different ages. This scenario occurs when using data from life course case-control studies, in which participants might come from different birth cohorts. Furthermore, because the disease might occur at different ages in adulthood, participants from the same birth cohort might not contribute to exposure during later life periods if disease onset is early, and this can also be incorporated using a hierarchy of parameters in Bayesian RLM.

An ‘Empty model’ in which the life course exposure variable is not included, is a scenario tested by some researchers.^49^ Such a scenario can be assessed in Bayesian RLM, by using a non-informative prior for the lifetime effect and the weights. If the exposure was not associated with the outcome, the posterior distribution would be very similar to the non-informative prior.

Bayesian RLM is not devoid of limitations. Because the overall effect of the life course variable is captured by a single parameter (lifetime effect), the model assumes that the exposure will have the same direction of association in all periods. However, this assumption is likely to be valid for most exposures. For example, betel quid chewing is unlikely to increase the risk of oral cancer in one period and reduce it in another without the influence of any other variable.

RLM assumes that there is no time-dependent confounding or effect measure modification. This is a common limitation of other strategies proposed for the investigation of life course hypotheses.^9–11^ One reason for such effect measure modification is the mediation effect by subsequent periods. Although techniques are available to estimate the mediation effect, recent reports recognize the difference in research questions addressed by the life course approach and causal modelling techniques such as marginal structural models.^50^^,^^51^ The relevance of life course investigations alongside more complex causal modelling techniques is also recognized.^50^^,^^51^

The accurate reconstruction of exposure histories across extended periods of participants’ lives is a challenge faced by researchers working in this field. For studies that do not have prospectively collected measures, techniques such as life grid-based interviews have proven useful to mitigate the issue to an extent.^24^^,^^25^ With the rapid growth in the use of ‘big data’ for epidemiological studies, accurate exposure histories might become easier to compile.

The adoption of Bayesian methods might be considered a daunting task by some researchers, because of the involvement of programming in MCMC software. We provide the code to fit Bayesian RLM in both RStan^52^ and SAS (SAS Institute, Inc., Cary, NC) in the Supplementary data, available at *IJE* online.

In conclusion, Bayesian RLM is a viable alternative for the investigation of life course hypotheses involving continuous exposures, as it allows for formal integration of prior knowledge, does not depend on *P*-values or variable selection procedures, and can provide direct inference on the probability of life course hypotheses.

## Funding

This work was supported by: the Canadian Institutes of Health Research [MOP 81172, MOP111207]; Ministère du Développement économique, de l'Innovation et de l'Exportation du Québec: Programme de soutien à la recherche (PSR), volet: Soutien à des initiatives internationales de recherche et d’innovation (SIIRI). M-C.R. was a recipient of a Career Award from the Fonds de Recherche du Québec – Santé. B.N. holds a Canada Research Chair in Life Course Oral Epidemiology. S.M. is the recipient of a doctoral scholarship from the Fondation Universitaire Armand-Frappier INRS and a Psychosocial Oncology Research Training (PORT) top-up award. R.H. is supported by the UK Medical Research Council [MC_UU_12019/2].

**Conflict of interest:** None declared.

## Supplementary Material

Supplementary Data
