# Supplementary material for: A Bayesian approach to investigate life course hypotheses involving continuous exposures
Source: Int J Epidemiol. 2018 Jun 14;47(5):1623–35. doi: 10.1093/ije/dyy107 (PMC6208282; doi:10.1093/ije/dyy107)
Supplement: Supplementary Data [file dyy107_suppl_data.pdf]

Supplementary material

Details of hierarchical Bayesian RLM used for real data example

Let X_{ti} denote the exposure measurement of $i^{th}\,(i = 1,2,\dots,N)$ participant at $t^{th}(t = 1,2,\dots.,T)$ time point. Also, $j\ (j = 1,2,\dots,J)$ denotes the participant's age group. In addition, y_{ij} denotes the oral cancer status of the participant and lx_{ij} denotes relevant life course exposure.

Likelihood:

$$lx_{ij} = \sum_{t=1}^{T} w_{tj} * x_{ti}$$

$$logit(p_{ij}) = \beta_0 + \delta_j * lx_i + \lambda * \ C_i$$

$$y_{ij} \sim Bernoulli(p_{ij})$$

$$\delta_j \sim Normal(\delta, \sigma)$$

$$w_{t,j} \sim Dirichlet(\alpha)$$

$$\alpha = \phi * \kappa$$

Priors:

$$\phi \sim Dirichlet(1,1,1)$$

$$\kappa \sim Poisson(10)T(6,)$$

$$\delta \sim Cauchy(0,2.5)$$

$$\sigma \ \sim Normal(0,1)$$

$$\beta_0 \sim Cauchy(0,10)$$

$$\lambda \sim Cauchy(0,2.5)$$

Parameterizing the Dirichlet distribution as expected value (ϕ) and strength of prior evidence (κ) allow us to easily state prior on the average weight function. Since there is no prior study in the field on betel quid chewing, we give the weak prior ($poisson(10), Dirichlet(1,1,1)$) for these hyperparameters. The codes for the above model in JAGS is available upon request from the corresponding author.

Stan code for Bayesian Relevant life course exposure model (RStan 2.14)

```
data{
      int<lower=0> N;         #Number of observations
      int<lower=0> T;         #Time points/ life periods of measurement
      int n_cons;             #Number of co-variates

      matrix[N,T] expMat;           #Matrix of exposure measures
      matrix[N,n_cons] conMat;      #Matrix of co-variates

      int<lower=0, upper=1> y[N];   #Binary outcome variable
      vector[T] Dalp;               #The parameters for dirichlet prior

  #Expected weights for critical period hypothesis
      vector[T] STC[T];

  #Reference vector of weights for accumulation period hypothesis
      vector[T] STA;
}
parameters{
      real alpha;             #Intercept
      real delta;             #Life-Time effect parameter
      vector[n_cons] lambda;  #Parameters for co-variates
      simplex[T] W;           #Weights
}
transformed parameters{
      vector[N] xb;
  #Linear combination
      xb = alpha + delta*(expMat*W) + (conMat*lambda);
}
model{
      alpha ~ cauchy(0,5);    #Prior for Intercept
      delta ~ cauchy(0,2.5);  #Prior for life-time effect

      for(i in 1:n_cons){
            lambda[i] ~ cauchy(0,2.5); #Priors for co-variates
      }
      W ~ dirichlet(Dalp);    #Prior for weights

      y ~ bernoulli_logit(xb); #Logistic likelihood
}
generated quantities{
      vector[T+1] EucDist;          #Euclidean Distances
      real exp_delta;         #Odds ratio of lifetime effect

      for(i in 1:T){
            EucDist[i] = distance(W, STC[i]);
      }

      EucDist[T+1] = distance(W,STA);

      exp_delta = exp(delta);
}
```

R code for simulation

```
# R version 3.3.2

#Loading libraries
      library(MASS)
      library(boot)

      library(rstan)
      rstan_options(auto_write = TRUE)
      options(mc.cores = parallel::detectCores())

#Setting seed for random number generator
      set.seed(28278)

#Sample sizes
      N1 <- 700
      N2 <- 1500
      N3 <- 3000

#Mean & SD of variables
      mu <- c(0,0,0)
      sd <- c(1,1,1)
      sd2 <- sd %*% t(sd)

#Correlation Matrix
      rho <- 0.7
      corMat <- cbind(c(1,rho,rho^2),c(rho,1,rho),c(rho^2,rho,1))
      corMat

#Covariance Matrix
      Sigma <- sd2*corMat

#Simulating three correlated exposure variables
      X1 <- mvrnorm(N1, mu=mu, Sigma = Sigma, empirical=TRUE)
      X2 <- mvrnorm(N2, mu=mu, Sigma = Sigma, empirical=TRUE)
      X3 <- mvrnorm(N3, mu=mu, Sigma = Sigma, empirical=TRUE)

#True Parameter values
#Overall effect
      beta <- 2

#Critical Period model = 3
      WC3 <- c(0,0,1)

#Pure Accumulation model
      WA <- c(1/3,1/3,1/3)


#Sensitive period model 1st period >2nd peiod >3rd period
      WSe <- c(0.75,0.20,0.05)
```

```
#Simulating Outcomes
#Critical period outcome
      y1C3 <- rbinom(N1, size=1, prob= inv.logit(beta*((X1 %*% WC3)[,1])))
      y2C3 <- rbinom(N2, size=1, prob= inv.logit(beta*((X2 %*% WC3)[,1])))
      y3C3 <- rbinom(N3, size=1, prob= inv.logit(beta*((X3 %*% WC3)[,1])))

#Accumulation outcome
      y1A <- rbinom(N1, size=1, prob= inv.logit(beta*((X1 %*% WA)[,1])))
      y2A <- rbinom(N2, size=1, prob= inv.logit(beta*((X2 %*% WA)[,1])))
      y3A <- rbinom(N3, size=1, prob= inv.logit(beta*((X3 %*% WA)[,1])))

#Sensitive period outcome
      y1Se <- rbinom(N1, size=1, prob= inv.logit(beta*((X1 %*% WSe)[,1])))
      y2Se <- rbinom(N2, size=1, prob= inv.logit(beta*((X2 %*% WSe)[,1])))
      y3Se <- rbinom(N3, size=1, prob= inv.logit(beta*((X3 %*% WSe)[,1])))

#Expected weight vectors
      StC <- cbind(c(1,0,0),c(0,1,0),c(0,0,1))
      StA <- WA
```

Supplementary Table 1. Posterior median of Euclidean distance from estimated weights to reference vectors of life course models

Sample size	True Life course Scenario	Reference vectors for standard life course scenarios*				
		**C1**	**C2**	**C3**	**A**	**S**
**700**	**C3**	1.29	1.30	**0.15**	0.67	1.09
	**A**	0.84	0.85	0.77	**0.10**	0.56
	**S**	0.31	1.13	1.22	0.53	**0.09**
**1500**	**C3**	1.38	1.38	**0.05**	0.77	1.18
	**A**	0.85	0.77	0.84	**0.07**	0.54
	**S**	0.30	1.12	1.24	0.54	**0.06**
**3000**	**C3**	1.36	1.36	**0.07**	0.75	1.16
	**A**	0.85	0.78	0.82	**0.06**	0.55
	**S**	0.34	1.08	1.23	0.52	**0.05**

*C1 = Critical period 1 (w1=1, w2=0, w3=0); C2 = Critical period 2 (w1=0, w2=1, w3=0); C3 = Critical period 3 (w1=0, w2=0, w3=1); A = Accumulation (w1=w2=w3=0.333), S = Sensitive period (w1=0.75, w2=0.20, w3=0.05). The lowest Euclidean distances are in bold.

Supplementary Table 2. Posterior mean and 95% credible intervals for absolute bias of parameter estimates under different life course scenarios and sample sizes

Parameters	Sample Sizes	Life course scenario (parameter values) Critical period	Accumulation	Sensitive period
**W1**				
	700	0.065<br>(0.004 – 0.154)	-0.017<br>(-0.134 – 0.099)	0.012<br>(-0.111 – 0.135)
	1500	0.021<br>(0.001 – 0.065)	-0.021<br>(-0.105 – 0.062)	0.017<br>(-0.068 – 0.104)
	3000	0.030<br>(0.002 – 0.075)	-0.024<br>(-0.084 – 0.036)	-0.007<br>(-0.065 – 0.053)
**W2**				
	700	0.054<br>(0.002 – 0.153)	-0.024<br>(-0.174 – 0.129)	-0.032<br>(-0.167 – 0.105)
	1500	0.019<br>(0.001 – 0.063)	0.036<br>(-0.073 – 0.146)	-0.019<br>(-0.118 – 0.076)
	3000	0.025<br>(0.020 – 0.072)	0.029<br>(-0.20 – 0.109)	0.012<br>(-0.060 – 0.080)
**W3**				
	700	-0.119<br>(-0.220 - -0.032)	0.041<br>(-0.077 – 0.157)	0.019<br>(-0.046 – 0.123)
	1500	-0.41<br>(-0.094 - -0.006)	-0.014<br>(-0.100 – 0.040)	0.002<br>(-0.047 – 0.076)
	3000	-0.055<br>(-0.106 - -0.013)	-0.005<br>(-0.066 – 0.055)	-0.005<br>(-0.047 – 0.050)
**Lifetime effect**				
	700	0.145<br>(-0.169 – 0.479)	0.086<br>(-0.228 – 0.417)	0.001<br>(-0.298 – 0.319)
	1500	0.052<br>(-0.158 – 0.270)	0.086<br>(-0.228 – 0.417)	0.061<br>(-0.151 – 0.280)
	3000	0.046<br>(-0.104 – 0.202)	-0.087<br>(-0.230 – 0.060)	0.066<br>(-0.087 – 0.223)

Supplementary Table 3. Comparing Bayesian RLM with the Structured approach using WAIC*

Approach	Model fit	Life course scenario WAIC (SE)		
		**Critical period**	**Accumulation**	**Sensitive period**
**Structured approach**	**C1**	888.39 (17.6)	783.39 (23.7)	679.72 (27.8)
	**C2**	818.98 (21.4)	736.73 (26.2)	789.28 (23.3)
	**C3**	**642.02 (28.4)**	769.93 (25.1)	879.25 (18.0)
	**A**	737.03 (24.9)	**678.84 (28.2)**	726.23 (26.4)
	**S**	643.80 (28.6)	682.22 (28.5)	**672.64 (28.5)**
**Bayesian Relevant exposure model**		**642.15 (28.3)**	**682.19 (28.1)**	**671.51 (28.2)**

*C1 = Critical period 1; C2 = Critical period 2; C3 = Critical period 3; A = Accumulation, S = Sensitive period. The lowest WAIC values are in bold.

Supplementary Table 4. Mean and Standard deviation of chew-years of betel quid chewing at three life periods (<20 years, 21-40 years, >40 years) by age group and case-control status

	Age groups									
	Below 40 years		41 to 50 years		51 to 60 years		61 to 70 years		Above 70 years	
**Life periods**	**Control**	**Case**	**Control**	**Case**	**Control**	**Case**	**Control**	**Case**	**Control**	**Case**
**< 20 years of age**	7.5 (10.6)	12.08 (16.8)	0.50 (0.1)	10.44 (16.3)	1.29 (2.6)	10.81 (17.0)	1.10 (3.3)	10.37 (17.7)	10.19 (20.0)	13.32 (18.3)
**21 to 40 years of age**	11.50 (4.9)	126.27 (97.8)	24.65 (29.3)	119.35 (103.1)	51.43 (60.8)	90.31 (69.4)	66.14 (65.7)	93.88 (79.0)	71.82 (77.6)	121.45 (117.1)
**> 40 years of age**	NA	NA	12.45 (2.7)	48.93 (48.6)	74.97 (52.5)	99.80 (66.8)	169.34 (161.3)	175.58 (117.1)	169.77 (176.8)	283.65 (160.4)

Supplementary Table 5. Mean and 95% credible interval for life period specific weights for chew-years of Betel quid chewing for different age-groups

	Age groups				
**Life periods**	**Below 40 years**	**41 to 50 years**	**51 to 60 years**	**61 to 70 years**	**Above 70 years**
**< 20 years of age**	61.7 (7.3 – 90.9)	53.4 (5.7 – 83.7)	55.1 (5.8 – 85.6)	56.5 (6.0 – 87.8)	53.8 (5.9 – 82.9)
**21 to 40years of age**	38.3 (9.1 – 92.7)	31.5 (6.9 – 72.8)	29.8 (5.9 – 72.4)	30.5 (6.7 – 72.4)	31.1 (6.8 – 73.2)
**> 40 years of age**	NA	15.1 (2.2 – 37.5)	15.1 (2.6 – 37.7)	12.9 (2.2 – 34.7)	15.2 (2.8 – 37.5)

Supplementary Table 6. Distribution of selected characteristics of HeNCe Life Study - India

	Control (n=350)		Case (n=371)	
	**n (%)**	**Mean ± SD**	**n (%)**	**Mean ± SD**
**Age**		60 ± 11		60 ± 11
**Sex**				
Female	167 (0.45)		154 (0.44)	
Male	204 (0.55)		196 (0.56)	
**Education**				
Low	182 (0.49)		267 (0.76)	
High	189 (0.51)		83 (0.24)	
**Material deprivation index**		20.44 ± 7.1		13.80 ± 6.4
**Pack-years of tobacco smoking**		12.18 ± 26.3		16.05 ± 28.2
**Liter of ethanol consumed**		0.18 ± 1.1		0.76 ± 3.0
**Betel quid**				
Never	306 (0.82)		97 (0.28)	
Ever	65 (0.18)		253 (0.72)	

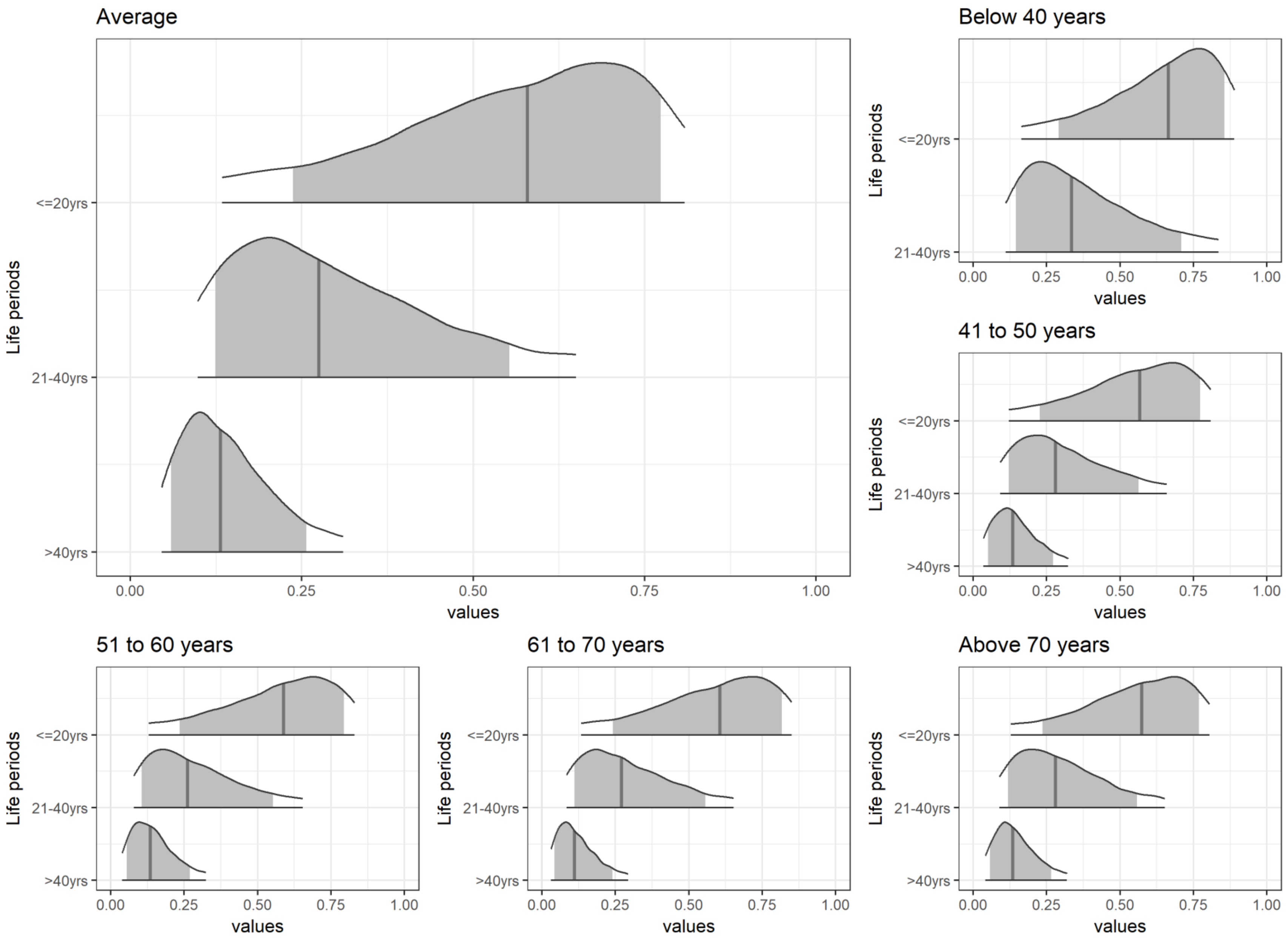

Supplementary Figure 1. Posterior densities of life period specific weights for chew-years of betel quid chewing for different age groups and average over groups

Supplementary Table 7. Life time effect of betel quid chewing on risk of development of head and neck cancer among ever users

	OR (95% Credible intervals)
Average	1.04 (1.00 – 1.15)
Age-group specific	
Below 40 years	1.04 (0.99 – 1.16)
41 to 50 years	1.07 (1.01 – 1.26)
51 to 60 years	1.05 (1.01 – 1.14)
61 to 70 years	1.04 (1.01 – 1.14)
Above 70 years	1.02 (1.00 – 1.04)

SAS code for Bayesian RLM

```
/*Bayesian Relevant Life Course Exposure model*/
/*Three life periods example*/
/*Binary outcome with logistic likelihood and no confounders*/


/*Using Marginal priors on weights*/

proc mcmc data=Data nbi=50000 nmc=100000 outpost=OutData
                    init = random monitor=(lw1 lw2 lw3 beta0 delta w);

       parms lw1 lw2 lw3 ;
       array w[3];
       w[1] = exp(lw1)/(exp(lw1)+ exp(lw2)+ exp(lw3));
       w[2] = exp(lw2)/(exp(lw1)+ exp(lw2)+ exp(lw3));
       w[3] = exp(lw3)/(exp(lw1)+ exp(lw2)+ exp(lw3));

       parms beta0 delta ;
       prior beta0 ~ cauchy(0,2.5);
       prior delta ~ cauchy(0,2.5);

       prior lw1 ~ beta(1,1);
       prior lw2 ~ beta(1,1);
       prior lw3 ~ beta(1,1);

       p = logistic(beta0+ delta*(w[1]*x1+w[2]*x2+w[3]*x3));
       model y ~ binomial(1,p);
run;



/*Using Joint multivariate prior on weights*/

proc mcmc data=Data nbi=50000 nmc=100000 outpost=DataOut
                    init=random diag=(mcse ess) propcov=quanew;

       array dal[3];
       begincnst;
              do i =1 to 3;
                     dal[i] = 1;
              end;
       endcnst;

       array w[3];
       parms w;
       parms beta0 delta;

       prior beta0 ~ cauchy(0,2.5);
       prior delta ~ cauchy(0,2.5);
       prior w~dirichlet(alpha=dal);

       p = logistic(beta0+ delta*(w1*x1+w2*x2+w3*x3));
       model y ~ binomial(1,p);
run;
```
